# Validation of a Ready-to-Use Lyophilized Kit for Labeling IL2 with ^68^Ga: A New Avenue for Imaging Activated T-lymphocytes in Tumor Microenvironment

**DOI:** 10.3390/jcm14165658

**Published:** 2025-08-10

**Authors:** Chiara Lauri, Valeria Bentivoglio, Michela Varani, Ilenia Cammarata, Giorgia Sartori, Silvia Piconese, Giuseppe Campagna, Alberto Signore

**Affiliations:** 1 Nuclear Medicine Unit, Department of Medical-Surgical Sciences and Translational Medicine, Sant’Andrea Hospital of Rome, “Sapienza” University of Rome, 00161 Rome, Italy; valeria.bentivoglio@uniroma1.it (V.B.); michela.varani@uniroma1.it (M.V.); giuseppe.campagna@uniroma1.it (G.C.); alberto.signore@uniroma1.it (A.S.); 2 Department of Translational and Precision Medicine, “Sapienza” University of Rome, 00161 Rome, Italy; ilenia.cammarata@uniroma1.it (I.C.); giorgia.sartori@uniroma1.it (G.S.); silvia.piconese@uniroma1.it (S.P.)

**Keywords:** ^68^Ga-IL2, PET/CT, activated T-lymphocytes imaging, kit development, shelf life, tumor microenvironment

## Abstract

**Background/Objectives**: Radiolabeled interleukin-2 (IL2) could allow for imaging activated T-lymphocytes in the tumor microenvironment (TME). The aims of this study were to assess the shelf life of a lyophilized kit containing THP-desIL2 to allow for the labeling of IL2 with ^68^Ga at room temperature and to test the in vitro binding of ^68^Ga-THP-desIL2 on different T-cell populations in order to determine which specific T-cell subset expresses the CD25 subunit of the IL2 receptor (IL2R). **Methods**: desIL2 was conjugated with THP and lyophilized. ^68^Ga labeling was performed and several quality controls, including HPLC, iTLC and SDS-PAGE, were carried out at different storage times (1, 3 and 6 months) and temperatures (4 °C and −80 °C). Moreover, flow cytometric analysis on different T-cell populations and the in vitro and competitive binding of ^68^Ga-THP-desIL2 were performed. **Results**: The lyophilized kit of THP-desIL2 was stable up to 6 months at −80 °C, preserving its sterility, integrity and acceptable values of labeling yield (51.80 ± 3.74%), radiochemical purity (>96%) and specific activity (5.59 ± 0.40 MBq/µg). Binding of ^68^Ga-THP-desIL2 on activated lymphocytes was specific and exhibited a low dissociation constant from IL2R on stimulated Tregs (Kd: 10^−9^–10^−10^ mol/L). **Conclusions**: We assessed the shelf life of a lyophilized kit containing THP-desIL2 for the easy labeling of IL2 with ^68^Ga at room temperature. The kit can be stored at −80 °C up to 6 months, thus facilitating the adoption of ^68^Ga-THP-desIL2 into clinical practice. ^68^Ga-THP-desIL2 showed high affinity and specificity for CD25 on activated T-lymphocytes, particularly Tregs, thus opening new opportunities for imaging immune cells trafficking in the TME.

## 1. Introduction

Our knowledge on the dynamic behavior of cells within their microenvironment, novel targets or pathways overexpressed in a given physio-pathological condition and the biological processes underlying many diseases have been radically improved by advancements in the molecular imaging field [1,2,3,4,5]. This has allowed for the possibility to develop targeted therapies and vaccines [6].

Given the critical role of the immune system in chronic inflammation, autoimmunity and cancer, targeting immune cells has emerged as an attractive strategy for diagnostic and prognostic purposes [1,2,3,4,5,7,8]. In particular, detecting, quantifying and characterizing the activated T-lymphocyte status would allow for the detection of molecular changes that precede clinical onset and radiological detection in a wide range of diseases, informing on disease activity and aiding in the selection of candidates for targeted therapies [8]. This would also address the urgent clinical need to monitor their dynamic trafficking during ITs in many autoimmune (AI) disorders, as well as in the tumor microenvironment [9]. Imaging immune cells can be accomplished by using antibodies or their fragments directed against specific targets expressed on T- or B-cells or the NK surface, or by using cytokines to track immune cells’ fate. Given their smaller molecular weight, the faster plasma clearance and the lack of immunogenicity, the imaging of cytokines offers several advantages compared to the use of whole antibodies or their fragments. Interleukin-2 (IL2) is one of the most studied cytokines over the past decades due its crucial role in promoting the proliferation, growth and differentiation of B-cells, NKs, monocytes, macrophages and oligodendrocytes [10]. These functions are mediated by the interaction of IL2 with its trimeric receptor expressed on activated T-cells. In particular, the interaction between IL2 and the CD25 subunit of the IL2 receptor (IL2R) has been extensively investigated as a reliable surrogate marker for imaging activated T-lymphocytes [8]. Several labeling methods with both gamma camera and positron emission tomography (PET) isotopes have been proposed over the past decades, demonstrating great diagnostic and prognostic potential in many preclinical and clinical studies [11,12,13,14,15,16,17,18,19,20,21,22,23]. The first preclinical studies with ^123^I showed excellent in vitro results and biodistribution [11]. Human studies were, therefore, conducted in patients with several inflammatory bowel diseases [12] and cancers [13,14], demonstrating the ability of labeled IL2 to quantify the presence of CD25+ cells (IL2R) and to monitor therapeutic efficacy. Nevertheless, due to the high costs of radioactive iodine, the use of ^123^I was soon replaced by ^99m^Tc. ^99m^Tc-IL2 was successfully used in patients with different inflammatory and autoimmune disorders [16] and in melanoma [17]. Despite the promising results, the labeling procedure with ^99m^Tc required approximately 4 h, thus limiting its use in routine practice.

The interest of radiochemistry research has, then, rapidly shifted in radiolabeling IL2 with PET isotopes (^18^F and ^68^Ga), which provides higher imaging quality compared to gamma camera [18,19,20,21,22].

First attempts were performed with ^18^F, but the procedure was complex, time-consuming and expensive, requiring cyclotron access [18,19,20]. ^68^Ga, due to its shorter half-life that perfectly aligns with the biologic half-life of IL2, could be the ideal isotope [22,23]. Nevertheless, the use of a chelating agent for ^68^Ga is necessary and often requires high temperature for the labeling, thus inducing IL2 denaturation and, consequently, the loss of its biological activity. Moreover, the conjugation of IL2 with a chelating agent is a time-consuming procedure requiring several purifications steps, which could limit its use in clinical routine. The availability of a ready-to-use lyophilized kit containing conjugated-IL2 would facilitate easy labeling with ^68^Ga at room temperature, avoiding the need to synthetize a new batch of conjugated-IL2 for each PET/CT scan.

We recently biochemically characterized different formulations of IL2 (desIL2 and human recombinant IL2) with different chelating agents, NODAGA and THP, identifying THP-desIL2 as the best combination to develop a lyophilized kit and to perform labeling at room temperature, obtaining a high labeling yield (LY), specific activity (SA) and radiochemical purity (RCP), and achieving excellent in vitro and in vivo results in mice experiments and in a phase I study in healthy volunteers. Moreover, we demonstrated that ^68^Ga-THP-desIL2 is able to bind, with high affinity, its specific receptor on activated lymphocytes [23].

The aims of this study were to assess the kit’s shelf life at different storage times (1, 3 and 6 months) and temperatures (4 °C and −80 °C), and the in vitro binding of ^68^Ga-THP-desIL2 on different lymphocyte subsets, to better understand which specific subpopulations overexpressing IL2R can be imaged by this approach.

## 2. Materials and Methods

### 2.1. Assessment of Lyophilized Kit’s Shelf Life

#### 2.1.1. Preparation of Lyophilized THP-IL2 Batches

Lyophilized THP-desIL2 batches were prepared following the procedure described in our previous study [23], and stored at 4 °C or −80 °C.

To determine whether different freezing times and storage temperatures influence the structure of the THP-desIL2 kit’s shelf life, quality controls (QCs) were performed before and after the radiolabeling with ^68^Ga by reconstituting the vials within 3 h (time 0) from their preparation and at 1, 3 and 6 months of storage.

Lyophilized vials containing 100 µg of THP-desIL2, stored at −80 °C or 4 °C, were reconstituted at each time point with 200 µL of dilution buffer. Following reconstitution, 50 µL (25 µg) were used for sodium-dodecyl-sulphate poly-acrylamide gel electrophoresis (SDS-PAGE), 50 µL (25 µg) were used for reverse-phase high-performance liquid chromatography (RP-HPLC) analysis and the remaining 100 µL (50 µg) were further diluted with 1 mL buffer solution for ^68^Ga-labeling.

#### 2.1.2. QCs Before Radiolabeling

SDS-PAGE analysis was performed using a Mini-PROTEAN^®^ Tetra Cell system (Bio-Rad Laboratories, Hercules, CA, USA) to assess the stability of the samples under various storage conditions. A 10–15% gradient SDS-polyacrylamide gel was used to analyze the molecular weight of the protein. Electrophoresis was carried out at constant voltage (typically 120 V) for approximately 90 min.

Following separation, proteins were visualized using GelCode™ Blue Safe Protein Stain (Thermo Fisher Scientific, Waltham, MA, USA). The electrophoretic bands of each sample were compared with native desIL2 at time zero, with an expected molecular weight range of 12.5–15.5 kDa.

RP-HPLC analysis was performed using a Shimadzu HPLC system (Shimadzu Corporation, Kyoto, Japan) with a Kinetex^®^ C18 column (5 mm diameter, 5 μm pore size and 250 mm length; Phenomenex, Torrance, CA, USA).

The elution was performed with water, with 0.1% TFA (Pump A), and acetonitrile, with 0.1% of TFA (Pump B), at a gradient of 0–10 min at 0–95% B, 10–15 min at 95% B and 15–18 min at 95–5% B, and a flow rate of 1 mL/min.

The column effluent was monitored for UV absorbance using a dual-wavelength spectrophotometric detector at 210 and 280 nm, and using an on-line detector for radioactivity (Flow-Count, BIOSCAN, Eckert & Ziegler, Wilmington, MA, USA), connected in series.

#### 2.1.3. Radiolabeling and QCs

Radiolabeling was performed using a GAIA synthesis module (Elysia-Raytest^®^, Liège, Belgium) equipped with a Germanum-68/Gallium-68 (Thema Solution, Verona, Italy) generator under the GMP condition, as previously described [23]. Briefly, 540 MBq (539.4 ± 34.6 MBq) of freshly eluted ^68^Ga-Cl3 solution was added to 50 µg (1 mL) of THP-desIL2, incubated for 15 min at room temperature and then purified by tC18 cartridge eluted with 100% EtOH in 0.9% NaCl.

The percentage of LY was automatically calculated by the GAIA^®^ synthesis module software (v2). The RCP (%) and SA (MBq/µg) were calculated by instant thin-layer chromatography (iTLC) with chromatography silica gel strips.

iTLC was performed using two different mobile phases to distinguish free isotopes, free THPs, colloids and ^68^Ga-desIL2, as follows:

1. MeOH/NH_4_OAc (1:1)

This system was used to assess RCP. The retention factors (Rfs) were as follows:

A. Free ^68^Ga: Rf ≈ 0.1;
B. Colloidal ^68^Ga: Rf ≈ 0.1;
C. ^68^Ga-THP-IL2: Rf ≈ 0.8–0.9.

The percentage of ^68^Ga-THP-desIL2 was calculated using the following formula:

%RCP = C/(A + B + C) × 100.

2. 5% NaCl/MeOH/25% NH3 (3:1:1)

This system was used to evaluate the presence of colloidal ^68^Ga.

The Rfs were as follows:

D. Free ^68^Ga: Rf ≈ 0.8–0.9;
E. Colloidal ^68^Ga: Rf ≈ 0.1;
F. ^68^Ga-THP-IL2: Rf ≈ 0.8–0.9.

The percentage of colloidal ^68^Ga was calculated as follows:

%E = E/(D + E + F) × 100

To determine the percentage of free ^68^Ga by subtraction, the following formula was applied:

%A = %(A + B) − %E

HPLC analysis of the radiolabeled compound was attempted by withdrawing 0.1 µg (20 µL), but it was not feasible since the sample was too diluted.

#### 2.1.4. Assessment of Sterility and Pyrogenicity of the Lyophilized Kit

At each time point, both before and after radiolabeling, small aliquots of reconstituted THP-desIL2 were injected into culture broths for anaerobic and aerobic bacteria to test sterility after 1 week. The presence of pyrogens was checked by a qualitative gel clot Limulus Amebocyte Lysate (LAL) assay before and immediately after the synthesis of the radiopharmaceutical.

### 2.2. In Vitro Binding on Activated T-lymphocyte Subsets

#### 2.2.1. Lymphocyte Sampling and Activation

Briefly, 10 mL of human blood from 3 different healthy donors were collected in a vial containing ACD anticoagulant (2 mL) and diluted with an equal volume of medium. The diluted blood was layered over 15 mL of Ficoll-Paque™ PLUS (GE Healthcare, Frankfurt am Main, Germany) and then centrifuged at 2500 rpm for 35 min. After centrifugation, the well-defined layer of mononuclear cells was collected and transferred to a vial, diluted with 2 mL of medium and then centrifuged at 2500 rpm for 15 min. The resulting pellet was diluted up to 10 mL with RPMI-1640 medium (GIBCO), plated in a Petri dish (100 mm, Corning, Corning, NY, USA) and activated in the presence of phytohemagglutinin-M (PHA-M, Sigma-Aldrich, St. Louis, MO, USA) at a concentration of 5 mg/mL.

After 48 h of incubation with PHA-M, cells were collected, counted with 0.4% Trypan Blue Solution (Thermo Fisher Scientific, Waltham, MA, USA) and centrifuged at 2500 rpm for 15 min to allow the isolation, through density gradient centrifugation, of a final concentration of 2.5 × 10^6^ vital human peripheral blood mononuclear cells (hPBMCs) per mL. hPBMCs where then analyzed by flow cytometry to evaluate the percentage of expression of CD25.

#### 2.2.2. Competitive Binding

We performed an in vitro evaluation of ^68^Ga-THP-desIL2 (freshly prepared) binding to hPBMCs in different conditions.

hPBMCs were isolated from healthy donors as previously described and plated in two different Petri dishes for 3 days at 37 °C. One Petri dish was treated with PHA-M (5 mg/mL) and the other one with PBS as control.

After 48 h, the cells were centrifuged, counted with Trypan Blue and suspended in 1 mL of RPMI-1640 medium (GIBCO) (5 × 10^5^/mL) supplemented with 10% fetal bovine serum.

Also, fresh hPBMCs were isolated from healthy donors, counted with Trypan Blue and suspended in 1 mL of RPMI-1640 medium (GIBCO) (5 × 10^5^/mL) supplemented with 10% fetal bovine serum.

In vitro binding tests were performed as follows:

A. Fresh hPBMCs + 50 µL (0.25 µg) of ^68^Ga-THP-desIL2 (35 µCi);
B. No PHA-M hPBMCs + 50 µL (0.25 µg) of ^68^Ga-THP-desIL2 (35 µCi);
C. PHA-M-activated hPBMCs + 50 µL (0.25 µg) of ^68^Ga-THP-desIL2 (35 µCi);
D. PHA-M-activated hPBMCs + 50 µL (0.25 µg) of ^68^Ga-THP-desIL2 (35 µCi) + 100 × THP-desIL2 (cold).

After an incubation of 30 min at 37 °C, the batches were centrifuged (4.000 rpm per 10 min) and the supernatant and pellet were collected. The activity from the supernatant and pellet was measured in an automatic gamma counter (2480 Wizard^2^, PerkinElmer, Waltham, MA, USA).

Three independent experiments were performed in triplicates.

#### 2.2.3. Phenotypic Analysis of Lymphocyte Subsets by Flow Cytometry

Flow cytometric analysis was used to evaluate the expression of the CD25 subunit of IL2R on specific hPBMCs’ subpopulations.

After collecting blood samples from 2 different healthy donors, the following populations were isolated as untouched cells by negative immunomagnetic cell sorting:

- B-lymphocytes were isolated from hPBMCs using the Pan B Cell Isolation Kit, human 130-101-638 for 10^9^ total cells (Miltenyi Biotec, Bergisch Gladbach, Germany).
- CD8+ T-lymphocytes were isolated using the CD8+ T Cell Isolation Kit, human 130-096-495 for 10^9^ total cells (Miltenyi Biotec).
- NK cells were isolated using the NK Cell Isolation Kit, human 130-092-657 for 10^9^ total cells (Miltenyi Biotec).
- Conventional (Tconv) and regulatory (Treg) CD4 T-cells were isolated using the CD4 + CD25+ Regulatory T Cell Isolation Kit, human 130-091-301 (Miltenyi Biotec).

To obtain high numbers of activated Tregs highly expressing CD25, Tregs were isolated and expanded in vitro for 14 days using the following reagents and related protocols: CD4 + CD25 + CD127dim/- Regulatory T Cell Isolation Kit II, human 130-094-775 for 1 × 10^9^ total cells, and Treg Expansion Kit, human 130-095-345 2 mL.

Purity and CD25 expression of the obtained populations were checked by flow cytometry with a combination of different monoclonal antibodies (mAbs) and reagents: viability dye (Fixable Viability Dye eFluor780, 65-0865-14, eBioscience, Carlsbad, CA, USA), CD25 (anti-human CD25 PE, 12-0259-42, eBioscience), CD19 (anti-human CD19 BV421, 562440, BD Biosciences), CD14 (in the dump channel, anti-human CD14 BV605, 564054, BD Biosciences), CD3 (anti-human CD3 FITC, 566783, BD Biosciences, San Jose, CA, USA), CD4 (anti-human CD4 BV510, 317444, Biolegend), CD8 (anti-human CD8 PeCy7, 344712, Biolegend, San Diego, CA, USA), CD127 (anti-human CD127 PeCy7, 351320, Biolegend), CD16 (anti-human CD16 AlexaFluor 647, 557710, BD Biosciences), CD56 (anti-human CD56 PerCP-Cy5.5, 560842, BD Biosciences) and FOXP3 (anti-human FOXP3 PerCP-Cy5.5, 45-4776-42, Invitrogen, Carlsbad, CA, USA).

Cells were centrifuged and re-suspended in PBS at an optimal concentration (1 × 10^7^/mL). Cells were washed and stained with Fixable Viability Dye (VD) eFluor780 (Thermo Fisher Scientific) in PBS for 30 min at room temperature. After washing, surface mAbs were added to the cells and incubated for 20 min at room temperature. To analyze FOXP3 expression, cells were fixed and permeabilized with the FOXP3/Transcription Factor Staining Buffer Set (Thermo Fisher Scientific) for 30 min at 4 °C and then intracellularly stained in permeabilization buffer for 30 min at room temperature with FOXP3 antibody. Cells were acquired with the LSR Fortessa cytometer (BD Biosciences) and analyzed with FlowJo software version 10.5.3.

#### 2.2.4. Kinetic Cell Binding Assay

Once analyzed by flow cytometry, the same subpopulations where plated in a Petri dish suitable for LigandTracer^®^ (Uppsala, Sweden), as described in the literature [24] for the kinetic binding assay.

To facilitate the attachment of the suspended cells to the Petri dish, the surface of the glass-bottomed culture dish was aseptically treated with 4 mL of BAM (Sunbright^®^ OE-040CS, NOF Corporation, New York, NY, USA) at the concentration of 2 mg/mL in Milli-Q^®^ water and incubated for 30′ at room temperature [24]. After washing, 4 mL of cells suspended in PBS (2.5 × 10^6^/mL) were plated for 40′ at room temperature, followed by washing with PBS to remove non-immobilized cells.

Freshly prepared ^68^Ga-THP-desIL2 was then added to the cells and counting was started for 60 min. The kinetic of cell binding over time, as well as the dissociation from cells (Kd), were evaluated. Raw counts per minute (cpm) of cells were corrected for background signal and for isotope decay.

#### 2.2.5. Immunoreactive Fraction (IRF) Assay

To assess the percentage of ^68^Ga-THP-desIL2 able to maintain affinity for its specific receptor (functional purity), the IRF assay was performed as follows: PHA-M-activated hPBMCs were seeded, with increasing concentration (from 0.1 × 10^6^/mL to 10 × 10^6^/mL), in vials containing the same amount of ^68^Ga-THP-desIL2 (3.8 kBq, 0.006 µg, and 24 × 10^10^ molecules). This IL2 concentration was chosen to give a saturation of 10 × 10^6^ cells, assuming 25.000 IL2R receptors per cell, as reported in the literature [25].

After 1 h of incubation at 4 °C, the vials were centrifuged and the supernatant was collected. This step was repeated after washing the pellet with 0.5 mL of PBS. Radioactivity associated with pellets and supernatants was then determined. Data were analyzed using Prism GraphPad software (v. 5.04) and an immunoreactive fraction (IRF) was calculated for each ^68^Ga-THP-desIL2 batch.

### 2.3. Statistical Analysis

Continuous variables are shown as mean ± standard deviation (SD) and 95%CI (Confidence Interval). A comparison between the groups in Table 1 and between fresh PBMCs, non activated PBMCs, activated PBMCs and PBMCs with an excess of desIL2 was performed with the Generalized Linear Mixed Model (GLIMMIX) method, considering Gaussian function as distribution. The normality of distribution of residuals was verified by the Shapiro–Wilk test and checking the Q-Q plot. Homoscedasticity was verified by checking the plot of residuals vs. the fitted plot. Post hoc analysis was performed by Tukey’s method. A *p*-value < 0.05 was considered statistically detectable.

The statistical analysis was performed by SAS v.9.4 TS Level 1M8 and JMP PRO v. 17.1 (SAS Institute Inc., Cary, NC, USA).

## 3. Results

### 3.1. QCs Before Radiolabeling of the Lyophilized Kit

**Figure 1:**
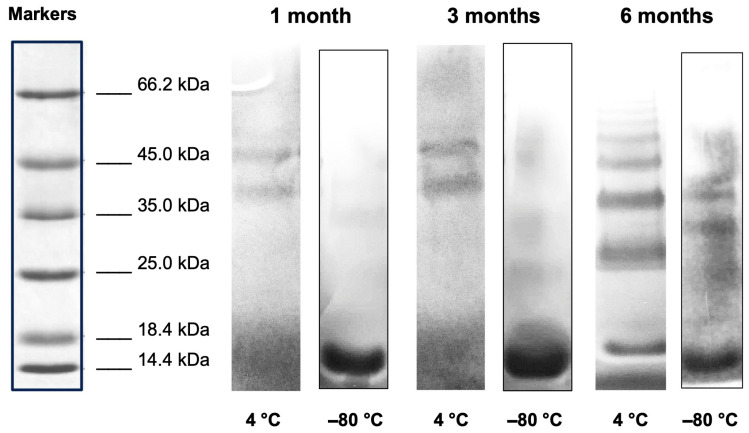
SDS-PAGE electrophoresis (8–16% SDS) of THP-desIL2 stored at 4 °C and −80 °C after 1, 3 and 6 months of storage.

SDS-PAGE results on THP-desIL2 stored at 4 °C showed the appearance of dimeric, trimeric and tetrameric complexes within 1 month of storage, whereas THP-desIL2 stored at −80 °C started to form a few dimeric complexes after 3 and 6 months, as shown in Figure 1.

The RP-HPLC analysis of the THP-desIL2 samples stored at −80 °C for 3 and 6 months showed a predominant peak eluting at approximately 7.1 min under the applied gradient conditions, as expected.

**Figure 2:**
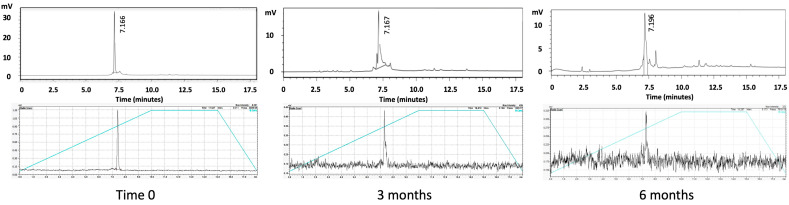
RP-HPLC of THP-desIL2 (upper panel) and radio-HPLC of ^68^Ga-THP-desIL2 (lower panel) at basal time and after 3 and 6 months of storage at −80 °C.

The UV absorbance at 280 nm of the THP-desIL2 samples confirmed the stability of the compound, with no significant shifts in retention time observed between the three time points (Figure 2, upper panel). No additional peaks nor significant degradation products were detected, suggesting minimal degradation or aggregation during storage.

RP-HPLC analysis of the THP-desIL2 samples stored at 4 °C showed a shift in the retention time at 1 month and the presence of multiple peaks, thus confirming the SDS results.

### 3.2. Radiolabeling and QCs

At the end of the radiolabeling process, a single dose contained 10 mL ^68^Ga-THP-desIL2 (25–30 μg, since approximately 50% of desIL2 remains in the tC2 column) in a 0.9% NaCl solution with 10% EtOH (dose 74–111 MBq; pH = 6–8).

Immediately after its preparation (time zero), ^68^Ga-THP-desIL2 showed an LY of 59.13 ± 2.58%, an RCP of 97.91 ± 0.45% and an SA of 6.29 ± 0.52 MBq/µg.

When stored at 4 °C, we observed a significantly lower LY at any time point (*p* < 0.0001) and SA (*p* < 0.0001 at 1 and 3 months and *p* < 0.0001 at 6 months) compared to THP-desIL2 stored at −80 °C, without significant modification on RCP (Table 1).

Limpid solutions were obtained after each labeling procedure at any time points.

### 3.3. Assessment of Sterility and Pyrogenicity of the Lyophilized Kit

Lyophilized powder of THP-desIL2 resulted sterile and non-pyrogenic (no gel clots were observed during the LAL test) at any time point, either before or after the radiolabeling.

### 3.4. Competitive Binding

**Figure 3:**
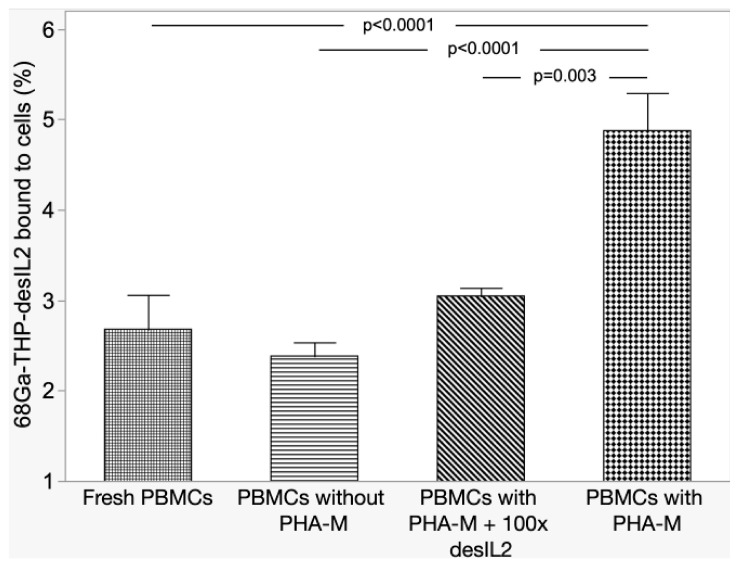
Results of competitive binding experiments.

^68^Ga-THP-desIL2 binding to PHA-M activated hPBMCs was significantly higher compared to non-activated or fresh hPBMCs, and, most importantly, compared to activated hPBMCs, with a 100 molar excess of cold THP-desIL2 due to the saturation of IL2Rs (Figure 3).

These results, therefore, underline the specific binding of ^68^Ga-THP-desIL2 on its receptor on activated lymphocytes.

### 3.5. Flow Cytometric Analysis and In Vitro Binding on Different Activated T-lymphocyte Subsets

Flow cytometry showed that 45 ± 5% of selected CD45+ cells after 48 h of activation express CD25.

**Figure 4:**
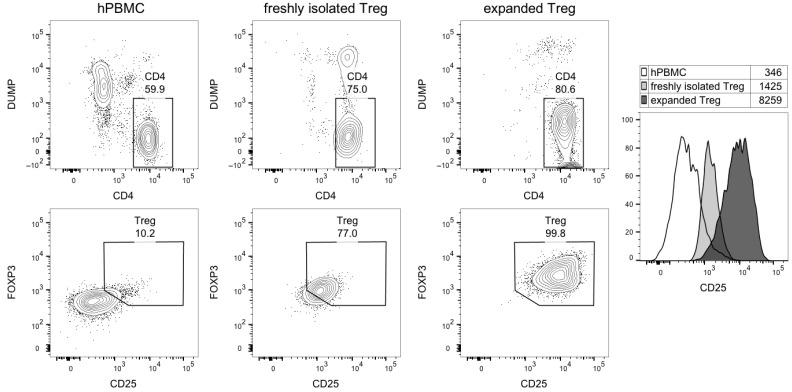
Flow cytometric analysis of Treg purity and CD25 expression. The cytograms show, in the upper line, CD4 vs. dump channel markers (dead cell dye, CD14/CD16/CD19/CD8), and, in the lower line, CD25 vs. FOXP3 expression in gated CD4 T-cells. Right, histogram overlay of CD25 expression in the indicated cell subsets; numbers shown the geometric mean fluorescence intensity.

This analysis also showed that CD25 was highly expressed on expanded Treg cells (Figure 4). Very low expression of CD25 was found on B-cells (<1%); thus, a kinetic binding assay was not performed (Table 2).

**Figure 5:**
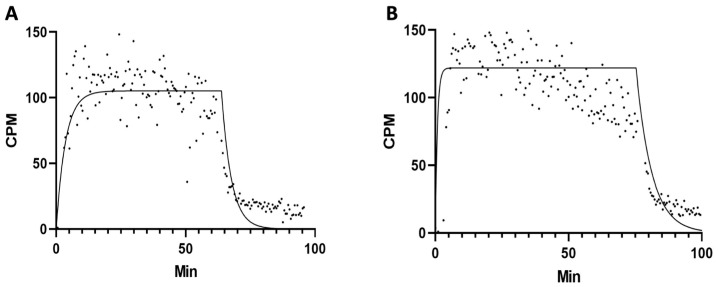
Representative binding assay of ^68^Ga-THP-desIL2 to purified human expanded Treg lymphocytes isolated from two different healthy donors ((**A**): Kd = 5.326 × 10^−9^; (**B**): Kd = 3.034 × 10^−9^).

Kinetic binding experiments confirmed previous data on PHA-activated hPBMCs (Kd 10^−9^–10^−10^ M) [23]. Similar results were obtained on stimulated Treg cells (Figure 5).

### 3.6. IRF Assay

IRF was performed on three different batches of ^68^Ga-THP-desIL2. Data analysis using GraphPad Prism software (v. 5.04) showed the mean IRF of ^68^Ga-THP-desIL2 was 85.94 ± 9.38%, (95%CI: 75.33% to 96.55%), indicating that a large proportion of the radiolabeled cytokine preserves its ability to bind IL2R after radiolabeling under the experimental conditions.

## 4. Discussion

Despite the labeling of IL2 that has been attempted over the past decades, with both single-photon-emitting and positron-emitting isotopes for imaging activated T-lymphocytes in many cancers and autoimmune disorders, labeling procedures have often been costly and time-consuming, thus limiting clinical applications. Nevertheless, given the solid proof-of-concept achieved so far with radiolabeled IL2, the interest in this approach has never faded.

In particular, labeling with positron-emitting isotopes has been explored by several groups [18,19,20,21,22,23]. ^18^F-labeled-IL2 has been used in a small cohort of metastatic melanoma patients to monitor the response to immune checkpoint inhibitor therapy. The results demonstrated the high blood pool activity of this radiopharmaceutical; however, it failed in detecting treatment-related immune responses. Moreover, immunohistochemistry analysis of T-cell subsets was available in only four patients, making it impossible to draw definite conclusions about the correlation between ^18^F-IL2 and CD25 expression in the TME [21].

Due to the high costs and the complexity of the labeling procedure, which requires a cyclotron [18,19,20], the attention has shifted toward labeling IL2 with ^68^Ga, which is cheaper, simpler and faster compared to ^18^F-labeling. One group, in particular, compared ^18^F-AlF-RESCA-IL2 and ^68^Ga-NODAGA-IL2, reporting good in vitro and in vivo results and preferential binding to activated hPBMCs for both radiopharmaceuticals, despite obtaining a low radiochemical yield [22].

We recently tested several formulations of IL2 (desIL2 and hrIL2) with different chelating agents (NODAGA and THP) to perform ^68^Ga-labeling at room temperature. ^68^Ga-THP-desIL2 achieved the best in vitro and in vivo results, with excellent biodistribution both in mice and in a phase I study, together with a favorable dosimetry and safety. Furthermore, we developed a ready-to-use lyophilized kit containing conjugated THP-desIL2 to streamline the labeling process, testing its shelf life over time at −80 °C [23].

Herein, we assessed the kit’s shelf life at different storage times (1, 3 and 6 months) and temperatures (4 °C and −80 °C) in order to facilitate its adoption in clinical practice and to verify the feasibility of storage at 4 °C in standard refrigerators. Moreover, we conducted an in-depth analysis of IL2R expression across different immune cell subpopulations in order to verify if ^68^Ga-THP-desIL2 can bind to CD25 expressed in different lymphocyte subpopulations.

This is the first study providing such detailed information while overcoming the previous limitations associated with labeling IL2 with PET isotopes by proposing the use of a ready-made lyophilized kit for ^68^Ga labeling at room temperature. Our results showed that the kit can be stored at −80 °C up to 6 months, maintaining its sterility, non-pyrogenicity, high RCP and significantly higher LY (51.80 ± 3.74 vs. 28.49 ± 2.05; *p* < 0.0001) and SA (5.59 ± 0.40 vs. 3.08 ± 0.22; *p* < 0.0001) compared to THP-desIL2 stored at 4 °C. Indeed, the SDS-PAGE results showed the appearance of dimeric, trimeric and tetrameric complexes within 1 month storage in vials stored at 4°C, whereas only a few complexes were observed in vials stored at −80 °C at 6 months. However, we suppose that they can disappear during appropriate sample reconstitution, dilution and radiolabeling, since no other forms but the monomeric can be observed by HPLC analysis after the reconstitution of lyophilized powder for the radiolabeling. Indeed, the SDS-PAGE samples were reconstituted with 0.5 mL of water, whereas, for radiolabeling, samples are reconstituted in 1 mL of labeling buffer. The availability of a ready-to-use kit would dramatically streamline the adoption of this novel imaging approach into clinical practice, avoiding the need of conjugation and further purification steps before each PET/CT scan.

The in vitro binding assay performed on PHA-M-activated hPBMCs, with and without the addition of a 100-fold excess of non-radiolabeled desIL2, demonstrated the specificity of ^68^Ga-THP-desIL2 binding to in vitro activated lymphocytes. Furthermore, all tested T-lymphocyte subsets and NK cells showed the binding of ^68^Ga-THP-desIL2 to CD25, although this receptor was variably expressed on different subsets (more on Tregs and CD8+ cells and very low on B-lymphocytes). It emerges that, in vivo, ^68^Ga-THP-desIL2 will detect prevalent Tregs and CD8+ cells, depending on their presence in different diseases.

Finally, we performed an IRF assay, which yielded a value of 85.9%. This indicates that a large proportion of ^68^Ga-THP-desIL2 retains the affinity of the native cytokine for its receptor, and that this functionality is also preserved after the radiolabeling.

These findings, together with data we previously obtained in vitro and vivo [23], and with the solid proof-of-concept acquired in the past decades on imaging IL2R, strongly support the use of ^68^Ga-THP-desIL2 for the identification and quantitation of CD25+ lymphocytes in tumors and in autoimmune diseases [26,27,28,29]. By visualizing the dynamic trafficking and interaction of immune cells and cancer cells, the use of ^68^Ga-THP-desIL2 could enable tailored therapeutic interventions, allowing clinicians to monitor immune therapies “in real time” and adjust approaches accordingly, thus opening new insights for imaging cancer.

## 5. Conclusions

We developed a lyophilized kit containing THP-desIL2 for the easy labeling of IL2 with ^68^Ga at room temperature. The kit can be stored up to 6 months at −80 °C, thus allowing us to dramatically streamline the adoption of this novel imaging approach into clinical practice, avoiding the need of conjugation and further purification steps before each PET/CT scan.

Given the high affinity and specificity of ^68^Ga-THP-desIL2 binding to CD25 expressed by activated T-lymphocytes, particularly Tregs, this approach would open new opportunities to image immune cells trafficking in the tumor microenvironment.

## Figures and Tables

**Table 1:** Results of the kit’s shelf life at different temperatures and storage times.

	Labeling Yield (%)		Radiochemical Purity (%)		Specific Activity (MBq/µg)	
	4 °C Mean ± SD (95%CI)	−80 °C Mean ± SD (95%CI)	4 °C Mean ± SD (95%CI)	−80 °C Mean ± SD (95%CI)	4 °C Mean ± SD (95%CI)	−80 °C Mean ± SD (95%CI)
Time 0 (Fresh)	59.13 ± 2.58 (52.73 to 65.54)		97.91 ± 0.45 (96.8 to 99.01)		6.29 ± 0.52 (5 to 7.58)	
One month	46.55 ± 0.68 (44.86 to 48.23)	58.18 ± 0.85 (56.08 to 60.29)	96.46 ± 0.57 (95.04 to 97.87)	97.43 ± 0.58 (96.00 to 98.86)	5.03 ± 0.07 (4.85 to 5.21)	6.28 ± 0.09 (6.06 to 6.51)
Three months	28.57 ± 0.79 (26.62 to 30.52)	57.15 ± 1.57 (53.25 to 61.05)	94.47 ± 1.09 (91.76 to 97.17)	96.20 ± 1.11 (93.44 to 98.95)	3.09 ± 0.08 (2.88 to 3.30)	6.17 ± 0.17 (5.75 to 6.59)
Six months	28.49 ± 2.05 (23.39 to 33.59)	51.80 ± 3.74 (42.52 to 61.08)	94.55 ± 2.65 (87.98 to 101.12)	96.48 ± 2.70 (89.78 to 103.19)	3.08 ± 0.22 (2.53 to 3.63)	5.59 ± 0.40 (4.59 to 6.60)

Labeling yield (%) and specific activity (MBq/µg): One, three and six month/s: 4 °C vs. −80 °C, *p* < 0.0001.

**Table 2:** Calculated bound molecules of ^68^Ga-THP-desIL2 per CD25+ cell in different lymphocyte subsets.

	Cells	CD25+ (%)	Positive Cells	Bound cpm	Bound Molecules	Molecules/Cell+	Kd
**PHA-M stim**	1,200,000	55	660,000	169	14,475,499,122	21,933	7.10 × 10^−10^
	400,000	43	172,000	97	3,055,904,961	17,767	4.43 × 10^−10^
**Treg**	3,000,000	86	2,592,000	122	75,551,668,782	29,148	5.33 × 10^−9^
	5,000,000	84	4,190,000	140	94,946,572,644	22,660	3.03 × 10^−9^
**Tconv**	28,000,000	3	896,000	110	18,849,855,178	21,038	8.49 × 10^−8^
	20,000,000	4	800,000	35	18,609,165,126	23,261	5.82 × 10^−8^
**CD8+**	10,000,000	12	1,200,000	363	31,664,418,911	26,387	9.19 × 10^−8^
	26,000,000	3	850,200	63	21,840,387,662	25,689	4.04 × 10^−8^
**NK**	4,800,000	5	240,000	87	6,444,763,338	26,853	1.81 × 10^−9^
	4,800,000	8	384,000	104	9,222,669,407	24,017	2.37 × 10^−9^

## Data Availability

Data are available upon request.

## Abbreviations

The following abbreviations are used in this manuscript:

IL2	interleukin-2
IL2R	interleukin-2 receptor
PET/CT	positron emission tomography/computed tomography
LY	labeling yield
RCP	radiochemical purity
SA	specific activity
hPBMCs	human peripheral blood mononuclear cells
